# Neurophenomenology revisited: second-person methods for the study of human consciousness

**DOI:** 10.3389/fpsyg.2015.00673

**Published:** 2015-05-29

**Authors:** Francisco A. Olivares, Esteban Vargas, Claudio Fuentes, David Martínez-Pernía, Andrés Canales-Johnson

**Affiliations:** ^1^Laboratory of Cognitive and Social Neuroscience, Psychology Department, Universidad Diego Portales, UDP-INECO Foundation Core on Neuroscience, Santiago, Chile; ^2^Pontificia Universidad Católica de Valparaíso, Valparaíso, Chile; ^3^Faculty of Psychology, Center of Argumentation and Reasoning Studies, Universidad Diego Portales, Santiago, Chile; ^4^Cognition and Brain Sciences Unit, Medical Research Council, Cambridge, UK; ^5^Department of Psychology, University of Cambridge, Cambridge, UK

**Keywords:** neurophenomenolgy, second-person methods, elicitation interview, descriptive experience sampling, double-blind interview, mutual circulation

## Abstract

In the study of consciousness, neurophenomenology was originally established as a novel research program attempting to reconcile two apparently irreconcilable methodologies in psychology: qualitative and quantitative methods. Its potential relies on Francisco Varela’s idea of reciprocal constraints, in which first-person accounts and neurophysiological data mutually inform each other. However, since its first conceptualization, neurophenomenology has encountered methodological problems. These problems have emerged mainly because of the difficulty of obtaining and analyzing subjective reports in a systematic manner. However, more recently, several interview techniques for describing subjective accounts have been developed, collectively known as “second-person methods.” Second-person methods refer to interview techniques that solicit both verbal and non-verbal information from participants in order to obtain systematic and detailed subjective reports. Here, we examine the potential for employing second-person methodologies in the neurophenomenological study of consciousness and we propose three practical ideas for developing a second-person neurophenomenological method. Thus, we first describe second-person methodologies available in the literature for analyzing subjective reports, identifying specific constraints on the status of the first-, second- and third- person methods. Second, we analyze two experimental studies that explicitly incorporate second-person methods for traversing the “gap” between phenomenology and neuroscience. Third, we analyze the challenges that second-person accounts face in establishing an objective methodology for comparing results across different participants and interviewers: this is the “validation” problem. Finally, we synthesize the common aspects of the interview methods described above. In conclusion, our arguments emphasize that second-person methods represent a powerful approach for closing the gap between the experiential and the neurobiological levels of description in the study of human consciousness.

## The Neurophenomenological Program

Francisco Varela in his article “*Neurophenomenology: a methodological remedy for the hard problem*” (Varela, 1996) proposed a novel research program for the study of human consciousness or lived experience by combining experimental methods of neuroscience with phenomenological methods of Western philosophy. “Phenomenology” has at least two different meanings in this context. First, it represents a research program for the study of human consciousness that includes phenomenology, introspection and meditation as its main methodologies (i.e., phenomenology as a *general* concept; Varela, 1996, p. 333). These are based, respectively, on the “reduction” and “suspension of the natural attitude” derived from the phenomenological tradition; on the attentional capacities derived from scientific psychology; and on meditation practices derived from Buddhist and Vedic traditions (Varela and Shear, 1999, p. 7). Second, phenomenology can be understood as a specific disciplined method for describing lived experience, first introduced by Husserl, and further developed by Merleau-Ponty and others (i.e., phenomenology as a *restricted* concept coming from the philosophical tradition). In the context of the neurophenomenological program, the notion of “Phenomenology” refers to the first sense (i.e., the general concept of phenomenology) which includes the Husserlian phenomenological tradition as one of its specific methodologies.

Given the importance of this topic for the rest of the article, it is worth drawing a strict distinction between the concepts of introspection and phenomenology (in its *restricted* conceptualization). Although both methods have existed for over a century, there are still contemporary studies that confuse the introspective and the phenomenological approaches, as has been alerted in several publications (Varela, 1996; Gallagher and Sørensen, 2006; Gallagher and Zahavi, 2008). This misinterpretation is even committed by very well known professionals of Cognitive Sciences, such as Dennett (1991), who confused the phenomenological method with introspectionism in his book “Consciousness explained” (Varela, 1996; Gallagher, 2000).

Nowadays two different procedures denominated “introspection” can be distinguished in the experimental research applied in Cognitive Sciences: introspection in a weak sense and in a strong sense (Gallagher and Sørensen, 2006). The weak concept of introspection is applied in those experimental designs where a person is required to report his or her own experience (in a verbal or behavioral response) when a specific stimulus is provided, e.g., a word, an image, a sound (Jack and Roepstorff, 2002; Price and Aydede, 2005). While the concept of weak introspection has been used for over a century in research focusing on the subjective experience of consciousness (Locke, 2009), introspection in the strong sense has been frequently confused with the phenomenological method. The reason for this misinterpretation is that the concept of introspection in the strong sense applies a method that makes the participant exclude any subjective interpretation from his or her experience and focus on their “pure” perception (Gallagher and Sørensen, 2006). Another characteristic of the strong concept of introspection is that the person, prior to the testing phase, is trained in this specific type of experiences that must be achieved. Initially, the researcher provides the participant with a number of the qualifying categories already operationalized by the investigator. Once the experiences in these categories are collected in verbal reports, the researcher transforms them into quantitative data, which could be compared with other third-person data (Gallagher and Sørensen, 2006).

On the other hand, there are a number of different variations of the phenomenological method. In the context of Varela’s work, the method incorporates three steps (Gallagher and Sørensen, 2006; Gallagher and Zahavi, 2008). In the first step, denominated as “suspending beliefs or theories about experience,” the interviewer raises open questions about the interviewee’s experiences. These questions lack any categorization or information that could bias the experience of the person. Through the method of open questions the interviewer aims for the individual to focus their attention on their own experience, reducing the number of possible interpretations (*epoché*). Through accomplishing the epoché, the person does not alter the veridity of the lived experience, but the interpretation on the phenomenon. This first step is similar to the concept of introspection in the strong sense. In the second step, called “gaining intimacy with the domain of investigation,” the interviewer gives the individual an insight into the study’s targeted experience. For that, the interviewer assists the person in exploring his or her experience in multiple ways (elaborated in the “Second-person method” section below). The task of the researcher is to enrich the conscious experience by guiding the session, which will allow the interviewee to more easily evoke the pre-reflective experiences. Thanks to this enrichment, the interviewee will have access to a new comprehension of his or her experience, i.e., to an intuition of his or her lived experience. While in the first and second steps the method is focused on the subjective experience, in the last step this changes. The third step in the neurophenomenological interview, termed “offering descriptions and using intersubjective validations,” is focused on externalizing the experience and sharing it with a community. This final step is of a great importance with respect to the scientific study of phenomenology. Depending on the scientific validity of this methodology, the results found in the interview might be replicated and accepted in the scientific community, or rejected based on the method’s inadequacy (this epistemological problem discussed below). In a nutshell, the descriptions that the interviewer collects are permanent properties that stay invariant during different analyses. They constitute a stable structure in the experience. Through this permanent feature, the same study could be repeated with other participants, and consequently confirmed or rejected in intersubjective validation. One important characteristic of this kind of validation is that the reports collected are not transformed into quantitative variables, which allows for keeping the data in its first-person experience state. This is an essential feature of phenomenological method. In the general context of the research, the phenomenological data (first-person), which was collected in a second-person interview, can be correlated with third-person data (behavioral response, neuroimaging, electroencephalography, EEG). Varela named this triple level of study as “mutual circulation” (Varela, 1996).

To summarize, introspection and phenomenology are different methods of studying the lived experience in first-person. Nevertheless, they are both part of the neurophenomenological program. The two methods are complementary in the first-person analysis. Varela expressed this notion in the following terms: “Phenomenology does share with introspectionism an interest in the reflective doubling as a key move of its approach to phenomena” (Varela, 1996, p. 338). From a procedural point of view, introspection is the first step in Varela’s phenomenology, showing the content of the lived experience. However, the introspective method is insufficient to explain the whole lived experience of a person. To complete this description, it is also necessary to integrate the phenomenological and the meditation methods. Just with the application of these three neurophenomenological methods the subjective experience can be comprehensively explained.

One of the goals of the neurophenomenology is to unify two different traditions, the neuroscientific experimental approach and the phenomenological approach (as a general concept) by integrating the lived, experiential data with neuroscientific data (Froese and Fuchs, 2012). Thus, neurophenomenology has to combine two distinct methodological procedures. On the one hand, the neuroscientific procedures furnish third-person “objective” data (e.g., fMRI, EEG) and, on the other, the phenomenological procedure provides first-person data, meaning reports of the observer’s lived experience. Just as there are many methods for obtaining third-person data, there are several means to acquiring data about a lived experience. These include -as described above- the phenomenological, introspective and meditative methods (Varela and Shear, 1999). To counteract the methodological differences between its constituents, the success of the neurophenomenological program requires a “necessary circulation” or communication between the first- and third-person accounts. In other words, neurophenomenology requires an integration of the third-person methods with all of the described first-person approaches (Varela and Shear, 1999).

Since its original conception, one of the main challenges for the neurophenomenological program is the creation of formal models for integrating both phenomenological and neurobiological accounts. The difficulty lies in establishing the correlation between local and global patterns of neural activity and their relationship with the phenomenal structure of the subjective experience (Lutz et al., 2002). Regarding the difficulty of generating models that link empirical and experiential data, Froese and Gallagher (2010) have suggested an intermediate step. They propose using agent-based modeling and mathematical simulation for developing formal models capable of integrating objective and subjective data. Another challenge in front of neurophenomenology has been the integration of extended and embodied aspects of human cognition. This issue has been addressed by investigating the relationship between cooperative patterns of neural activity and ecological (Colombetti, 2014; Desmidt et al., 2014), environmental (Beaton, 2013), and embodied/social aspects of the phenomenal experience (Lutz and Thompson, 2003; Froese and Fuchs, 2012). Thus, the problem of constructing a “bridge” between phenomenology, neurobiology and the environment finds its place as one of the main challenges of the neurophenomenological program.

During the last decade, several researchers have explicitly incorporated first-person phenomenological data in their third-person experimental protocols, exemplifying neurophenomenological practice (Lutz et al., 2002; Garrison et al., 2013; Petitmengin et al., 2013). In one of the first and most influential examples, Lutz et al. (2002) correlated phenomenological reports, reaction times and dynamical analysis of brain activity EEG. Thus, EEG activity was recorded during the presentation of a point pattern with depth information called an auto-stereogram. Each participant had to press a button when they achieved to clearly observe the three-dimensional figure and make a report of their experience. According to their first-person accounts, different phenomenological categories (or clusters) were identified regarding the level of participant’s preparation at the time the three-dimensional figure was perceived. According to these phenomenological clusters, EEG recordings were classified and patterns of brain functional connectivity (i.e., gamma-band phase synchrony) were computed. Their interest on first-person data was based on the hypotheses that the variability of the brain response after the perception of the three-dimensional figure could be generated by mental fluctuations attributable to the attentional state of the subject, or spontaneous thinking processes, or cognitive strategies on the task, among other possibilities (Gallagher, 2010, p. 24). Their “descriptive strategy”—a set of phenomenological techniques used for improving first-person accuracy of reportability of internal experiences (Bayne, 2004, p. 352)—can be described in two steps:

First, experimental subjects were trained. Training consisted of a set of practices for increasing the attentional sensitivity over actions and internal states that are related to the present experience (Thompson et al., 2005). Specifically, training was based on the practice of the *epoche* (Gallagher, 2010) which consists of the suspension of all previous judgments about the external world in order to attend to the experience itself; it is a description not of the content of *what* subject knows, but rather of *how* the experience happens. For Lutz et al. (2002), training consisted of improving subjects’ performance in the perceptual discrimination task as well as in the accuracy of their experiential reports. Participants were asked open questions between trials, which directed their attention toward their own mental processes. It was hypothesized that their responses could account for the between-subject variability in objective task performance by revealing differences in strategies employed, distractions, attentional states, and the like. Unlike traditional paradigms that deploy averaging techniques in order to attenuate the high variability of neural activity when interpreted as “noise,” Lutz et al. (2002) studied these “noisy” signals as representative of subjective parameters by taking into account the descriptions given by the participants after every trial.

Second, participants’ experiential reports were given after each trial and were organized into “phenomenological clusters.” “Phenomenological clusters” represented categories of trials, established based on first-person reports, that captured subjective variability during the task. Each cluster then permitted dynamical analysis of brain activity (Gallagher, 2010). For each phenomenological cluster, subcategories based on the state of preparation felt by the participants prior to stimuli presentation were identified. For instance, in the “steady readiness” cluster subjects reported that they were “well prepared” when the imaged appeared on the screen. Thus, phenomenological clusters gave richer and multidimensional information about the structure of conscious experience by accounting not just for the behavioral data (reaction times) but also for the neural correlates associated with conscious experience. In particular, prior to the presentation of the visual stimulus, they observed a large-scale pattern of phase synchrony in the frontal brain region in prepared subjects but not in unprepared subjects. Second, the degree of phase scattering recorded in the back electrodes was also modulated by the degree of preparation, i.e., the lower the degree of preparation the greater dispersion phase. Third, an earlier large-scale pattern of phase synchrony in prepared subjects (300 ms) than in the no-prepared subjects (600 ms) for the motor response.

As this example shows, successful neurophenomenology requires collecting large amounts of first-person data. This may appear to be a straightforward task if it would be enough to just ask the participant what they were experiencing during the trials. However, this is not the case at all; a *disciplined* observation of the experience is required. For instance, it would be a mistake to assume that being aware of a particular experience is sufficient for providing adequate verbal description (Bockelman et al., 2013). Furthermore, it is required that participants are able to attend the experience (introspection) while still being able to suspend their beliefs or previous judgments (phenomenological reduction), to avoid (as far as possible) bias or contamination of first-person reports. A powerful way to address these problems is to structure first-person data collection using *interviews* that are mediated by a trained person able to help the participants with describing their experiences accurately, that is, an interviewer or mediator with the “attitude” for reporting first-person data. This approach is known as the *second-person perspective* (Varela and Shear, 1999). Furthermore, the mediator does not take a neutral position but rather examines the participant from an empathic stand for investigating an experience *in common*. Just like an ethnographer is not simply interested in compiling data from a community as a neutral observer but in apprehending a way of living, a mediator may employ a specific strategy for apprehending first-person lived experiences. Varela and Shear (1999) claim that “a mediator is eccentric to the lived experience (...) but nevertheless takes position of one who has been there to some degree, and thus provides hints and further training” (p. 8).

In this paper, we conclude that second-person methodologies represent a promising set of tools for studying human consciousness in the context of the neurophenomenological program. We first describe second-person methodologies available in the literature for analyzing subjective reports, identifying specific constraints on the status of the first-, second- and third- person methods. Second, in the discussion we analyze two experimental studies that explicitly incorporate second-person methods for traversing the “gap” between phenomenology and neuroscience. On the other hand, we evaluate the challenges that second-person accounts face in establishing an objective methodology for comparing results across different participants and interviewers: this is the “validation” problem. Finally, we conclude by showing the common aspects of the interview methods described above, in order to provide an overview of the general properties of this important methodological approach.

## Second-person Methods

In this section we describe three second-person interview methods. Although these methods were not initially considered as part of the neurophenomenological program, we think they could be incorporated in the program if they meet to a possible extend the following three features. First, second-person methods require to be evaluated in the “mutual circulation” between the first, second and third person (Varela and Shear, 1999). For instance, the interviewer’s and interviewee’s description of the lived experience could be correlated with objective third-person data (Froese, 2013). Second, the acquisition of second-person data requires the mediation of a qualified interviewer. By a qualified interviewer we mean an emphatic mediator who could guide the interviewee in the process of becoming aware of their lived experience. Finally, the data collected by the interviewer should be used for computing correlational analyses with the neurobiological data, These three features are tightly related to neurophenomenology’s “mutual circulation” between the phenomenological and the neurobiological data. Importantly, these three characteristics we propose here represent an aspiration for the neurophenomenological program. They could be partially or completely met depending on the limitations imposed by the complexities of specific experimental designs. These three features that we propose here for the second-person neurophenomenological method are similar to the three steps in the Varela’s work. The first two features, to which the *epoché* of the phenomenological method should be added, attempt at developing the report of the lived experience. The third characteristic allows for the neurophenomenological analysis.

### Descriptive Experience Sampling

Descriptive experience sampling is an introspective method developed by Russ Hurlburt during the 1990s for observing and describing inner experiences—such as thoughts, feelings and visualizations—and their perceptual components (Hurlburt, 2011). “Experience” refers to the attention-, stimulus-, and context-dependent contents of consciousness at a particular time (Hurlburt and Schwitzgebel, 2011). For instance, now I am attending to the letters I am reading (what is “outside” the subject), then to my thoughts about what I am reading (what is “inside” the subject), then I become aware of those thoughts when suddenly a memory irrupts or I start attending to a sound of the environment. Thus, inner experience, or simply “experience,” refers to everything that is “directly present,” what appears “directly before the footlights of consciousness” (Hurlburt, 2009, p. 157; for a discussion of the concepts see Hurlburt and Schwitzgebel, 2007, p. 15). In this setting, the term “pristine experience” describes what is already happening *before* attending to the reflection or observation, as when we get lost in our thoughts until something appears “out there” and grabs our attention without making us lose the flux of experience at any moment (Hurlburt and Akhter, 2006). Therefore, the aim of DES is to achieve faithful and informative apprehension of the interviewee with their experience, and of the interviewer with a description of the interviewee’s experience. Altogether, this approach provides a phenomenologically valid and experimentally useful description of a particular experience at a particular moment.

The methodological procedure of DES consists of equipping the participant with an electronic device (a “beeper”) which emits a sound that participant can hear through headphones while occupied by his usual activities (Hurlburt and Heavey, 2004). Previous training has ensured that participants are adept in attending to their ongoing awareness (the pristine experience) at the moment of the “beep” (Hurlburt and Heavey, 2004, p. 13). The beeper sounds 5 or 6 times per day and its function is to facilitate the phenomenological report in minimizing retrospection, and to reduce the perturbation of the ongoing experience. Importantly, the function of the beep is to select an experience *randomly*, thereby avoiding bias associated with intentionally selecting moments. Thus, each random “beep” commands the subject to attend to his inner experience at that particular moment, and to register (by written or verbal recording) the features of that particular experience. Finally, 24 h after collecting experimental samples, an iterative process of interviews takes place (Hurlburt, 2009). According to Hurlburt, the role of the iterative process of interviews is to allow for increasing the salience of pristine experience:

The direct contribution of pristine experience is likely to decrease within each interview because the influence of reconstructions during the interview is likely to outpace the bracketing of presuppositions, even if genuine progress is made in bracketing presuppositions (i.e., the removal of parts of the interview contaminated by presuppositions or assumptions) and clarifying communication. However, if genuine progress is made in bracketing presuppositions and clarifying communication, the direct contribution of pristine experience is likely to increase across interviews because of the refreshment by the new pristine experience at each step (Hurlburt, 2009, p. 165).

All in all, the DES iterative process represents a useful method for obtaining first-person reports by facilitating “pristine” accounts of experience. An important feature of DES is its second-person character, arising from the essential role of the interviewer in mediating participants’ ability to describe their phenomenological experience and in thus validating their reports.

### Elicitation Interview

The elicitation interview (EI) is a technique developed by Pierre Vermersch at the end of the 1970s (Vermersch, 1994, 1999, 2009) designed to guide the interviewee to redirect her attention to specific aspects of her experience and to precisely describe them (Petitmengin and Lachaux, 2013; Valenzuela-Moguillansky, 2013).

Originally, Vermersch developed this technique in the context of his study of the cognitive processes involved in learning, particularly in problem resolution. Vermersch was interested in the procedural aspect of problem resolution, meaning the trajectory and the strategies that the person used while performing a certain task and not only in reaction times or success rates, which were the measures primarily used at that time. Thus, he designed a questioning technique that focuses on the physical or mental actions involved in performing a given task. In this sense, the EI focuses on the “how” of the experience rather than on the “why” or “what” (Petitmengin, 2006). For example, when I read, normally my attention is turned toward the content of *what* I read and not toward the processes involved in *how* I make sense of what I read. The latter normally stays implicit. The EI is oriented to access to this type of knowledge, hence its name, to make explicit what was hitherto implicit.

According to Petitmengin et al. (2007), the need of a technique to access the procedural aspects of our experience resides in the fact that, despite the intuitive belief that being aware of our own experiences is a fast and straightforward process, for us it is normally difficult to attend to the procedural aspects of our own lived experiences. Thus, one of the roles of the interviewer in this technique is to help the interviewee to sustain and stabilize his or her attention to such aspects.

Since its creation, the use of the EI spread to other contexts, particularly to the one of cognitive science and neuroscience (e.g., Petitmengin et al., 2007; Braboszcz, 2012; Valenzuela-Moguillansky et al., 2013; Gould et al., 2014; and see interview examples Maurel, 2009). Just like brain activity is recorded to obtain neuro-electric data in search for regularities of specific neuro-dynamic structures, Petitmengin (1999, 2006) and Vermersch (1994) have proposed collecting detailed interview data in search for regularities of specific “pheno-dynamic” structures.

The procedure of the EI could be described in terms of the following steps: (a) selecting a particular experience; (b) evoking the experience; (c) inquire into the temporal unfolding of the experience, or *diachronic* dimension; (d) inquire the experiential aspects that characterize each moment of the experience or *synchronic* dimension (Petitmengin, 1999, 2006; Petitmengin et al., 2013).

Step (a) relies on accurately selecting an experience in a particular moment. It would depend on the specific protocol whether the cognitive process associated with the experience under investigation is easily reproducible or not. If so, the researcher could devise a protocol enabling the interviewee to carry out the process here and now, and later through questioning to describe how he went about performing the process (Petitmengin, 2006).

Step (b) refers to evoking a past experience, which is to “*recalling a given experience as if re-enacting it*” (Valenzuela-Moguillansky, 2013, p. 340) in order to emphasize the retrospective contact. The experience may either have recently occurred (i.e., a cognitive task the participant has just performed) or it could have happened several years before, in which case the aim is for the past experience to be manifested in the present to the extent possible. Thus, the interviewer has to guide the interviewee toward an “embodied position” (Valenzuela-Moguillansky, 2013), in which they stand on the spatio-temporal context of his experience (the when, where and with whom). This embodied position facilitates the association of sensations and emotions by means of recalling episodic or autobiographical memory (Petitmengin et al., 2013).

In order to determine the effectiveness of evoking the past experience, Petitmengin (2006) used certain verbal, paraverbal and non-verbal “hints” which were indicative of the “strength” of a particular past experience. Thus, some ocular movements were indicative of the sensory register employed by the evocate state. For example participant’s fixating sight on the horizon often represents attending to their inner voice. In other cases, information about the “strength” of the past experience was derived from speech rhythm or vocal intonation. These were gestures the interviewer attended to because they went beyond the contents of speech. Instead, they represented a switch to pre-reflective consciousness of the experience to which the interviewee was attending.

In Step (c) the interviewer redirects the attention of the interviewee toward the procedural aspects of the experience (Valenzuela-Moguillansky, 2013). Once the interviewee is in a state of evocation, the interviewer helps the interviewee to describe the development of the experience using as axes of questioning her mental or physical actions. The interviewer guides the interviewee through the continuous flux of moments that characterize the unfolding of the experience or its diachronic dimension. The participant’s attention is directed to *how* a particular experience happened in time instead of what that experience was about or *why* did it happen. The set of questions asked by the interviewer are called “empty content” questions (Petitmengin and Bitbol, 2009) since they are aimed at the structure of the experience rather than the content of the experience.

The interviewee is encouraged to take time exploring their experience and to try and avoid judgments or preconceptions about their experience or about the interview process, in order to maximize their attention on the procedural aspects of the experience itself. The participant is often asked to clarify certain non-verbal gestures or vague words (Petitmengin, 2006).

Step (d) refers to the identification of the characteristics of each moment of the given experience. According to Valenzuela-Moguillansky (2013), once a sequence of actions has been established, the interviewer can guide the interviewee’s attention toward more subtle levels of the experience, such as bodily sensations, mode of attention, the characteristics of different sensorial modalities, etc. If we make an analogy of a participant’s experience as a movie, a continuum of the frames of a cinematographic film, the diachronic dimension would be the sequence, frame by frame, of that continuum (Petitmengin, 1999). In contrast, a synchronic dimension cannot be described by successive temporal relations but rather by the emotional tone, the mobilized attention, and the sensory registers employed in the evocation. In this synchronic or spatial configuration of the interviewee’s experience, their attention is directed to the structural aspect of the experience. For instance, describing the spatial features of what they “become aware” of, “the shape of the mental image,” “the position of the image [which] appears at a given distance, in a given direction, with a given size, etc. (Petitmengin, 2006, p. 251).”

Once a set of interviews is completed, comes the analysis of the interviews. In a nutshell, the analysis of the interviews is a process of abstraction that aims to identify the generic structure of an experience. The first step is the identification of the sequence of actions and points of articulation between different stages or “phases” of the experience, which has been called the *diachronic structure* of the experience. The second step is the identification of the experiential categories that characterize each phase of the diachronic structure of the experience, which has been identified as the *synchronous structure* of the experience.

Then, through a process of comparison, comes the identification of the invariants of the diachronic and synchronic structures of the experiences of a group of persons. From these invariants *the generic structure* of a given type of experience is built (Petitmengin, 1999, 2006; Vermersch, 2009).

To sum up, the EI permits accessing specific aspects of a particular experience. It employs a technique of questioning aimed at stabilizing interviewee’s attention and redirecting it toward the procedural aspects of the experience. In this way theoretical (i.e., the experimenter conceptualizing what the experience was) or representational (i.e., the participant only describing instead of reliving the experience) accounts of the experience is avoided. The analysis of the interviews allows building a generic structure of the experience identifying its “pheno-dynamic,” which can be used to incorporate the experiential aspects of a cognitive phenomenon to its neuroscientific study.

### Neuro-linguistic Programming

Neuro-linguistic programming was developed in the 1970s by Bandler and Grinder (1975) as a method for effective communication and personal development. It has been applied in personal coaching (Linder-Pelz and Hall, 2007), clinical therapy (Heap, 1988), among others (Sturt et al., 2012; Pishghadam and Shayesteh, 2014; and for a review of NLP studies see Witkowski, 2010). NLP studies how we experience the world through our senses and how we process consciously or non-consciously our percepts. NLP is further interested in the neural correlates of these processes (i.e., the *neuro* aspect). In addition, the programming aspect of NLP is concerned with how language is used for signifying the world (i.e., the *linguistic* aspect), and how people represent their experience by generating regular linguistic “patterns” (i.e., the *programming* aspect; Linder-Pelz and Hall, 2007).

The major contributions to the development of the NLP come from the work of Vermersch (1994) and Petitmengin (2006) who reconsidered several theoretical aspects of psychophenomenology for the study of experiences (Tosey and Mathison, 2010). For this reason, the NLP should not be considered as a method independent of the EI but rather as a complementary tool for the study of consciousness in its “pre-reflective” aspect (Mathison and Tosey, 2009, p. 193).

The NLP offers the opportunity to incorporate in the interview the experiential dimension of language in its verbal and non-verbal forms. Thus, Tosey and Mathison (2010) proposed a three step interview considering the following aspects: enabling evocation; identifying a “meta-model”; and eliciting sensory details. “Enabling evocation” refers to the introspective exercise of guiding the interviewee toward a state of evocation in which they re-create a past experience as if it is happening in the present (similarly as in the EI). The next step consists of investigating language patterns, that is, how the participant configures a taxonomy of syntactic structures, namely, the “meta-model” (Bandler and Grinder, 1975). The meta-model is indicative of the patterns that configure the interviewee “map of the world.” In other words, creating the “meta-model” involves investigating the configuration of the interviewee’s syntactic statements in order to reveal the underlying structure of the manner in which they signify their world.

The meta-model has been used for constructing questions in order to investigate “modal operators,” which according to Tosey and Mathison (2010) are “*words that define the mode in which an action is to be carried out, such as ‘will’, ‘can’, ‘may’, ‘might’, ‘won’t’ and so on*” (p.37). Thus, in a prototypical study, the questions and instructions could guide the participant to attend to the patterns of their language and to the changes in their internal representations by the usage of one word or the other. For instance, Tosey and Mathison (2010) describe a study that was looking to elucidate the distinctive subjective experience of the operators *could* and *will*. Participants had to think of an activity they were intending to do without specifying the activity. Then, they were asked to think about the activity in such a way that they would be aware of and able to re-present the event to themselves. They were instructed to report the changes of those internal representations using the modal operators *could* and *will*. Particularly, the interviewer asked a participant: “(...) *and if I say ‘You could do it?*’; [the participant replied:] *That’s much gentler. The kinaesthetic is more relaxed, it’s em…. The external auditory effect is one of support, so it’s my choice… the internal, the picture is soft, still clear, but soft.*” (p. 20).

The last step corresponds to the elicitation of sensory details, conceptualized as internal sensory representations and their submodalities, as explained by Tosey and Mathison (2010): “*re-creations of experience as internal representations, a pre-verbal level of cognition where the senses were engaged in the subjective re-presentation of experience, lived or imagined*” (p. 23). Examples of representational systems are the visual, auditory, kinesthetic, olfactory and gustatory dimensions, among others investigated during the interview. On the other hand, the submodalities are sensory registers that contain properties belonging to each modality; for instance, a register of the auditory modality is the volume of the participant’s voice (and its changes), its rhythm, intensity, etc.

NLP, as a second-person method, is based on the necessary contribution of the interviewer for directing participant’s attention toward the “inner search” of their own experience; for inviting the participant in a sort of “exchange between situated individuals” (Depraz et al., 2003, p. 81); and for creating questions that allows for the investigation of a particular experience. We have to stress that, to our knowledge, the application of NLP has not been considered experimentally in the context of the neurophenomenological program. The closest instance of using NLP in the study of phenomenological experience is the research conducted by Andreas and Andreas (2009) regarding the experiential distinction between perceptual positions; as well as the theoretical work of Barsalou (2008) about grounded cognition.

## Discussion

In the following section, we take a more analytical stance regarding the role of the second-person methods in the study of conscious experience. First, we put forth some qualifications regarding the status of the first-, second- and third-person methods that we believe are important to bear in mind. Second, we analyze two experimental studies that explicitly incorporate second-person methods for gaining access the “gap” between phenomenology and neuroscience. Third, we analyze the challenge that second-person accounts face in establishing an objective methodology for comparing results across different participants and interviewers, i.e., the validation problem. Finally, we conclude by synthesizing the common aspects of the interview methods described above.

### The First-, Second-, and Third-person: Some Qualifications

We would like to briefly clarify that the distinction of persons that neurophenomenology makes can be done not only by the mode of accessing lived experience, but also by distinguishing how many persons are involved in an investigation about consciousness. Thus, we have to notice that when we study consciousness, there is always someone who investigates and someone who is being investigated. In a first-person investigation there is, obviously, only one person. In this case, the researcher and the person being investigated are the same person. Thus, here the *subject* and the *object* of the knowledge are one only. In other words, if the researcher wants to investigate some aspect of consciousness, they should undergo the experience by themselves. In a second-person investigation, on the other hand, there are two persons, one that investigates (the interviewer) and another one that is being investigated (the interviewee). Finally, in the third-person research, there are at least three persons, one that is under investigation, and one or more (a community) that investigates.

If we fail to take into account the last point, we could mistakenly conclude that the first-person method is the only faithful method for accessing lived experience since no mediation is needed. This is an idea that comes from the ancient philosophers such as Aristotle for whom knowledge occurs in God, defined as the intellection that “intellects itself” (Aristotle, 1958). The idea is further developed, for instance, in Descartes (2008), for whom the most evident knowledge is the thinking or awareness of himself. However, we have to bear in mind that first-person accounts require *discipline* in the context of neurophenomenology. As we have seen, it is not enough for the participant to describe his lived experience. They could be imprecise due to lack of attention or training, or to being biased by previous beliefs. This is why the mediation of the third and, in particular, the second person is highly relevant for studying the first-person accounts.

Some authors, such as Dennett (2011), propose that the status of first-person data should be considered as a kind of belief of the participant until they can be objectively tested by a third-person study (e.g., in a neuroscientific context). This implies that the only method that reaches the truth of the phenomenon is the third-person. Nevertheless, the neurophenomenological approach assumes “the lived experience” as data which are reported in first-person (Thompson, 2011). But it must be taken into account that the phenomenological report is influenced by the level of the person’s training in attending to their thoughts, feelings and body or their ability to report the experience. This is the reason why it is necessary to add the studies in second-and third-person. The scientific explanations in each of the three persons have their own validity in the research, and neither poses a better level of explanation than the other. In fact, if applied in an explanatory circularity procedure, they allow for strengthening and improving the knowledge of the object of study. Although the debate between heterophenomenology and neurophenomenology is far away from the scope of this review, we advocate a neurophenomenological approach for the study of the lived experience where the phenomenological data are not considered as mere beliefs.

There are some meaningful ways to summarize the distinction between DES, EI and NLP. As Hurlburt (2011) puts it, “DES aims to make the visible memorable, EI aims to make the invisible visible” (p.70); in other words, DES try to make the visible visible, not assuming there are pre-reflective aspect of the experience and capture the directly experienced with fidelity enough; theoretically opposite to EI declare to find the source of the visible or that is not directly experienced. And the core methodological difference as Petitmengin and Bitbol (2011) put it, “EI is interested in what the subject *does* to apprehend his experience in the course of an interview [while] DES focuses exclusively on what happens “*before* the beep”—“pristine experience”—and not on what the subject does after the beep to describe his experience”(p.96).

Although the NLP has contributed methodologically to the development of the EI (Mathison and Tosey, 2009) because it shares some features with the introspectionist tradition, the theoretical structure of the NLP incorporates representational notions that are in conflict with the “embodiment” nature of the phenomenological program, e.g., with the “radical embodiment” stance (Thompson and Varela, 2001). However, in this manuscript we have considered the methodological aspect of the NLP as a second-person interview method rather than its explanatory account as a representational theory.

### Testing the “Mutual Generative Constraints”

As we mentioned in the introduction section, the interplay between neuroscientific knowledge and phenomenological accounts is at the core of the neurophenomenological program. Despite the advances in the interview protocols, the incorporation of second-person methods in the neuroscientific study of consciousness is still scarce (Froese et al., 2011). However, here we show two potential examples of how second-person methods “could” casts a new light on the “gap” between these two (neuroscientific and phenomenological) levels of description of consciousness.

#### The DES in the Study of Mind Wandering

An example of the application of the DES is given by the work of Christoff et al. (2009) who investigated the *default network* during mind wandering. The study was concerned with whether brain regions associated with the default network were activated in the same time window as when the mind was wandering and moving away from the task. Participants were asked to sample their experience at random intervals in order to determine whether they were mind wandering and whether they were aware or unaware of their mind wandering. They showed an activation in the medial prefrontal cortex in association with both subjective reports and behavioral measures. Also, when participants were unaware of their own mind wandering, both default and attentional networks were strongly activated. This study provided direct evidence for the neural recruitment associated with mind wandering by combining experience sampling with the tools of cognitive neuroscience. One limitation of this study is that the level of introspection required was minimal as the design lacked a theoretical and methodological appreciation of principled first- and second-person methods (Froese et al., 2011). Despite this limitation, the finding of default network recruitment in association with subjective experience sampling helps validate the use of reports in the study of consciousness.

#### The EI in the Study of Epileptic Seizures

A paradigmatic case of the interplay between EI and neuroscience is the study of Petitmengin et al. (2007) who investigated the prodromic symptoms in the subjective experiences occurring before a seizure, which corresponds to the preictal that precedes epileptic seizures. Since the preictal neuro-electrical changes are correlated with changes in the subjective experience of epileptic patients, the authors showed how preictal/epileptic anticipation represents an example of the mutual dependence between the neuro-dynamic and pheno-dynamic analyses (Petitmengin, 2010).

In Petitmengin et al.’s (2007) study, EI application consisted of a progression of the steps of the interview process. First, they asked patients to remember and retain a seizure experience that had generated vivid sensations, images or sounds. Then, they guided the patient toward a concrete evocation of that particular preictal experience, by helping them rediscover the experience until they feel as if “reliving” it. During the reliving moment, the interviewer had to attend to a group of precise verbal, paraverbal and non-verbal clues, which indicated the patient was really going back to a past experience. Once the evocation was stable, they asked appropriate questions that helped the patient turn their attention toward the various registers of his pre-reflective experience (e.g., emotional tone, visual, auditory registers) in order to describe accurately the experience that they were “reliving” (Petitmengin, 2010).

The significant contribution of this study did not consist only of the detection and comparison of neurological and phenomenological data, but also of establishing the “*mutual generative constraints*” (Varela, 1999) between the two. The concept of mutual constraints is in the core of the idea of enriching both phenomenological and neurological data by generating mutual restrictions between them. This idea is consistent with the finding of a neurodynamic structure preceding seizures, namely, the “preictal neuro-electric desynchronization” and, reciprocally, of a corresponding phenodynamic structure of the preictal experience.

In conclusion, both the DES and the EI contributed for the detection of certain specific neuronal configurations that were not predefined but rather emerged when both levels of explanation—the neurological and phenomenological—are considered in the analyses. Despite the theoretical and methodological limitations described above, these studies showed the fertility of the second-person methods when phenomenological accounts are permitted to guide the neurodynamic analysis.

### Second-person Methods and the Problem of Validation

It is however, of great importance to seriously consider to what extent the verbal descriptions of conscious experience resulting from second-person techniques valid for the scientific community. In particular, the challenge is to establish an objective method for comparing results across different participants and interviewers. This is challenging because the interview methods are prone to bias in several respects (see Froese et al., 2011). However, this is not an exclusive problem of the second-person methodologies since the first- and third-person methods are equally prone to be biased (Froese, 2013). For example, first-person accounts require training since the description of the experience can be obscured by the judgments or belief of the person who lives it. The interviewer, on the other hand, could influence the interviewee answers. Finally, the third-person (the scientific community) could introduce a bias by invalidating first-person accounts (Varela et al., 1992, p. 12).

Nevertheless, and with the aim of reducing the bias in the methodologies in second-person, the three steps in Varela’s approach have the purpose of increasing scientific validity through a procedure based on objectivity. As Gallagher and Zahavi (2008) point out, the aim of the phenomenological method is to achieve an objective procedure for the research of the subjective experience, which is a basic requirement in the scientific methodology. However, it is necessary to clarify that the model of objectivity proposed by Varela’s second-person method is different to those just based on third-person methods. This difference lies in the lack of equalling objectivity with a reductionist model of quantitative measurement and in advocating a method of intersubjective validation. Through the intersubjective model some invariant structures are identified in the interview of different individuals. This persistent report among subjects does not suffer from subjective interpretation of their experiences or subjectivity of the investigator. Therefore, the neurophenomenological method can guarantee the replication of other studies that investigate the same kind of phenomenon.

In Addition, Varela proposed to solve the problem of validation of the subjective experience by mutual circulation, that is, an explanation in first-, second-, and third-person. The lived experience (first-person) and (second-person) should be reciprocally validated against the collective experience of the scientific community (third-person). In line with this idea, the double blind interview (DBI) is, to our knowledge, the only method that explicitly proposes a solution for the validation problem.

#### The Double Blind Interview

Froese et al. (2011) proposed the DBI as the first step toward an objective measure of the fidelity of introspective accounts. In the context of the validation problem, a response to the challenge would be to determine whether participants are able to improve their introspective performance by employing second-person methods in the context of classical experimental paradigms. Thus, the DBI is conceived as a method for calibrating and validating other interview methods. The authors exemplify their proposal by using an experimental paradigm of crowded visual displays. In the words of Froese (2013):

Subjects are briefly presented with an array of visual stimuli and then asked to report what they have seen. It has been found that, although subjects report that they consciously experienced the whole crowded visual display (and they can indeed report any one of the items if appropriately primed), if left to their own devices they can subsequently report only a small subset of about four items. The methodological question is to what extent this retrospective blindness can be overcome with the guiding help of a suitably trained interviewer. Ideally the interviewer should not have seen the crowded display that was presented to the subject. This helps to avoid introducing implicit biases into the interview process, which is why we proposed to call this particular kind of second-person method the “Double Blind Interview” (p. 673).

Also, the DBI attempts to measure and standardize both the interviewer’s (second-person) and interviewee’s (first-person) introspective skills by incorporating an objective measure (i.e., a score) for establishing the authenticity of the reports published in the context of the scientific community (third-person). In Froese et al. (2011) words:

A score for facilitated recall (calculated on the basis of an interviewer’s ability to facilitate recall for a number of different participants, or on the basis of an interviewee’s recall ability to be facilitated by a number of different interviewers, or some combination of the two) could be introduced as an explicit requirement for publishing verbal reports of lived experience. In this way readers would be enabled to objectively assess the level of introspective skill which played a part in the generation of the reports, and hence their reliability and authenticity (p. 59).

In its first conceptualization, the DBI was proposed as a method of comparing two interview methods available at that time, i.e., the EI and the DES. However, it was not exempt of criticism by the authors of these methods. For instance, on the side of the EI, Petitmengin and Bitbol (2009) expressed reservations regarding the proposition of using external performance criteria to evaluate the reliability of interview-based measures of lived experience. In the DES camp, Hurlburt (2011), despite supporting the efforts for developing methods that validate phenomenological accounts, claims that validating the DES using DBI methods results impossible (p. 76). Hurlburt (2011) claims that DES cannot be validated under objective measurements since it represents a practice-based approach. In his view, DES can only be validated by assessing the internal coherence of the experience under investigation (i.e., the different internal aspects of the lived experience) rather than by objective measures.

It is important to notice that the DBI proposal has not been tested experimentally so far. However, according to Froese (2013) and despite the authors who previously doubted DBI’s validity, a similar method has been tested by Petitmengin et al. (2013) by incorporating the EI into the “choice blindness” paradigm (Nisbett and Wilson, 1977; Johansson et al., 2006). In this paradigm, participants are presented with a pair of portraits of women and are asked to choose which one they prefer. This procedure is repeated for 15 trials. After six of the trials subjects are handed their chosen photo and asked to explain their choice; but in three of these trials they have actually been secretly handed a non-chosen photo. Interestingly, the results showed that with the help of the EI participants detected that their choice had been manipulated in 80% of the trials, compared to only 33% when the choices were not followed by an EI. This result indicates that even if our awareness seems to be poor, it is still possible to consciously access our decision-making processes when our attention is directed toward its constitutive dimensions (Petitmengin et al., 2013).

In conclusion, the DBI emerges as the first explicit attempt for solving the validation problem in the context of the neurophenomenological program. Although it has not been tested experimentally so far, other similar methods have shown evidence for the direct efficacy of a second-person approach to the measure of conscious experience.

## Conclusions: The Role of the Second-Person in the Interview Methods

Finally, we would like to conclude by summarizing the common roles of the second-person (i.e., the interviewer) in the methods described above. Despite the theoretical and methodological differences between the interview methods, several interdependent aspects between second-person methods can also be found.

### Increasing the Ability of the Interviewee for Describing Lived Experience

In the DES, this can be noticed during the “expositional interview” performed by a skilled interviewer for helping to “bracket the natural attitude” (in Husserl’s terms) or, in other words, for suspending participant’s judgments about the nature of their experiences. By systematically repeating this procedure for a number of days, the EI aims to train the interviewee in becoming aware of their lived experience in such a way that they report it more accurately. Similarly, the contribution of the interviewer in both the EI and DBI is to facilitate the detailed reliving of a specific past experience by re-evoking it, and by directing the attention of the participant toward previously unattended or forgotten aspects of the experience. Finally, in the NLP framework, the interviewer investigates the cognitive and affective maps of the experience, trying to identify the different dimensions of the conceptual structure of the experience and to make the interviewee aware of these during the interview process.

### Validating the Mutual Circulation between the First- and Third-person Accounts

Regarding the well-known difficulty of the interviewee at the moment of specifying, recognizing and categorizing their own internal states, the role of the interviewer as a guider and “catalyst” of the process of becoming aware is crucial for all of the methods described above. On the other hand, the first-person requires a disciplined training in order to describe their experience since reporting own experiences is not very common in daily life. Also, for an untrained participant, their primary experiential accounts can be indistinguishable from their secondary cognition (i.e., judgments, beliefs justifications). Thus, second-person methods possess an eliciting potential for faithfully describing first-person accounts as a measure of the ability to describe lived experience.

### Attending the Accounts in Different Levels of Description

A crucial feature of the interview methods is that they allow the interviewer to examine the interviewee experience on different levels. In particular, first-person accounts can be described in both their verbal and non-verbal aspects (Petitmengin, 2006). Thus, visual and kinesthetic non-verbal indicators are especially relevant during the interview. For instance, the interpretation of gestures, the location of eyes in the space, or the movements that follow the verbal accounts, could bring non-explicit information about specific aspects of the interviewee’s experience. In addition, the interviewer could calibrate the non-verbal indicators performed by the interviewee by interpreting them, and thus improve both the introspective skills of the participant as well as their own interview skills (Bockelman et al., 2013).

In conclusion, the present article has emphasized that second-person methods represent a powerful but still underappreciated approach for closing the gap between the experiential and the neurobiological levels of description in the study of human consciousness.

### Conflict of Interest Statement

The authors declare that the research was conducted in the absence of any commercial or financial relationships that could be construed as a potential conflict of interest.
